# Supplementary material for: A Null B-Ring Improves the Antioxidant Levels of Flavonol: A Comparative Study between Galangin and 3,5,7-Trihydroxychromone
Source: Molecules. 2018 Nov 26;23(12):3083. doi: 10.3390/molecules23123083 (PMC6321095; doi:10.3390/molecules23123083)
Supplement: Supplementary file 1 [file molecules-23-03083-s001.zip › molecules-393628-SI/Suppls/Suppl. 1 Dose response curves.pdf]

Supplement 1: Dose response curves

Null *B*-ring Improves the Antioxidant Levels of Flavonol: A Comparative Study between Galangin and 3,5,7-Trihydroxychromone

Xiaojian Ouyang [1,2,†], Xican Li[1,2,†,*], Wenbiao Lu[1,2], Xiaojun Zhao[1,2], and Dongfeng Chen [3,4,*]

1 School of Chinese Herbal Medicine; Guangzhou University of Chinese Medicine, Guangzhou 510006, China. E-mails: 931504113@qq.com (X.O.);

2 Innovative Research & Development Laboratory of TCM; Guangzhou University of Chinese Medicine, Guangzhou 510006, China.

3 School of Basic Medical Science, Guangzhou University of Chinese Medicine, Guangzhou, China, 510006

4 The Research Center of Basic Integrative Medicine, Guangzhou University of Chinese Medicine, Guangzhou, China, 510006. E-mail: chen888@gzucm.edu.cn (D.C.)

* Correspondence: lixc@gzucm.edu.cn (X.L.); lixican@126.com (X.L.); chen888@gzucm.edu.cn (D.C.) Tel: +86-20-39358076; Fax: +86-20-38892690

† These authors contributed equally to this work.

Note: This Supporting Information file provides the original data of Table 1 in the main text. All underlined data are mentioned in Table 1 in the main text.

Superoxide anion (•O_2^-) scavenging assay

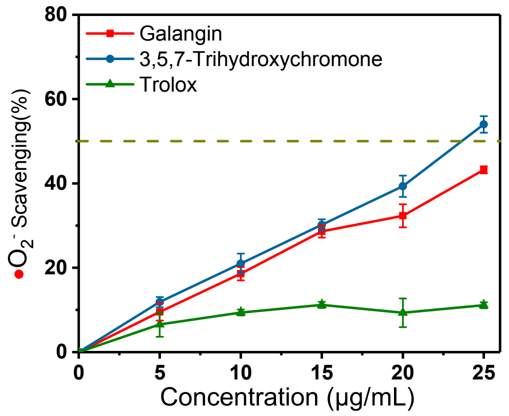

Figure S1: The dose response curves of galangin and 3,5,7-trihydroxychromone in •O_2^--inhibition assay. Each value is expressed as mean ± SD (n = 3).

Table S1. The comparison of IC_{50} values of galangin and 3,5,7-trihydroxychromone and positive control in •O_2^- scavenging assay.

	**Mean±SD μg/mL**	**Mean±SD μM**
Galangin	29.4±1.2	108.7±4.4 [a]
3,5,7-Trihydroxychromone	24.4±0.9	125.4±4.9 [b]
Trolox	1243.6±39.5	4968.4±157.9

IC_{50} value was defined as the concentration of 50% superoxide anion radical inhibition and calculated by linear regression which was analyzed by Origin 6.0 professional software. Means values with different superscripts in the same column are significantly different (p<0.05).

DPPH•-scavenging assay

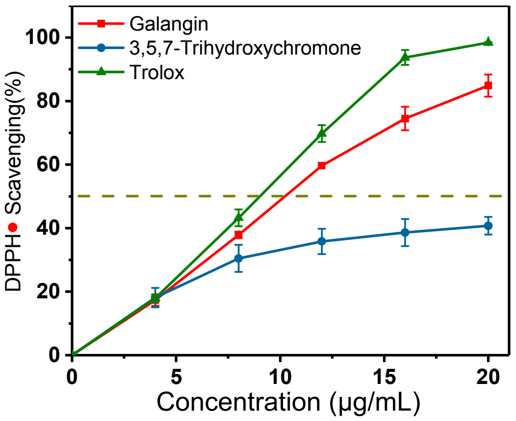

Figure S2: The dose response curves of galangin and 3,5,7-trihydroxychromone in DPPH•-scavenging assay. Each value is expressed as mean ± SD (n = 3).

Table S2. The comparison of IC_{50} values of galangin and 3,5,7-trihydroxychromone and positive control in DPPH•-scavenging assay.

	**Mean±SD μg/mL**	**Mean±SD μM**
Galangin	2.7±0.1	10.2±0.3 [a]
3,5,7-Trihydroxychromone	26.0±4.2	134.1±21.4 [b]
Trolox	9.2±0.3	36.9±1.0

IC_{50} value was defined as the concentration of 50% superoxide anion radical inhibition and calculated by linear regression which was analyzed by Origin 6.0 professional software. Means values with different superscripts in the same column are significantly different ($p<0.05$).

PTIO•-scavenging assay

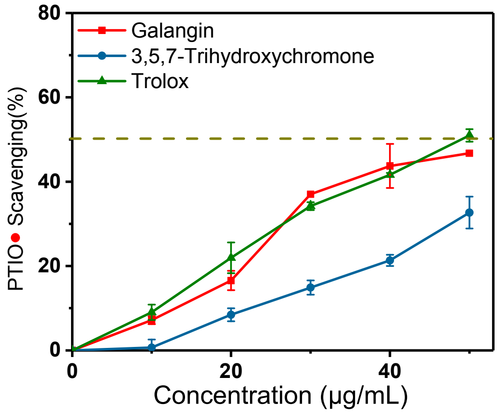

Figure S3: The dose response curves of galangin and 3,5,7-trihydroxychromone in PTIO•-scavenging assay. Each value is expressed as mean ± SD (n = 3).

Table S3: The comparison of IC_{50} values of galangin and 3,5,7-trihydroxychromone and positive control in PTIO•-scavenging assay.

	Mean±SD μg/mL	Mean±SD μM
Galangin	2.7±0.1	176.7±24.3 [a]
3,5,7-Trihydroxychromone	26.0±4.2	395.1±33.3 [b]
Trolox	9.2±0.3	186.3±7.2

IC_{50} value was defined as the concentration of 50% superoxide anion radical inhibition and calculated by linear regression which was analyzed by Origin 6.0 professional software. Means values with different superscripts in the same column are significantly different ($p<0.05$).

Fe^{3+}-reducing power assay

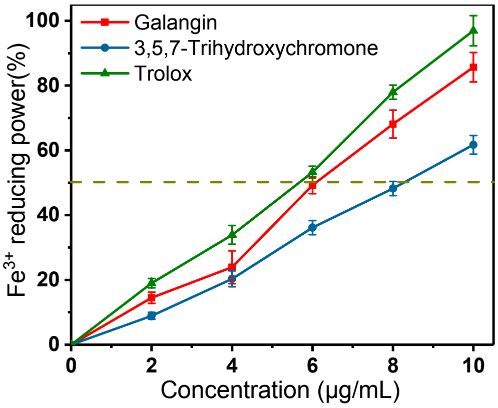

Figure S4: The dose response curves of galangin and 3,5,7-trihydroxychromone in Fe^{3+}-reducing power assay. Each value is expressed as mean ± SD (n = 3).

Table S4: The comparison of IC_{50} values of galangin and 3,5,7-trihydroxychromone and positive control in Fe^{3+}-reducing power assay.

	Mean±SD μg/mL	Mean±SD μM
Galangin	6.2±0.4	22.9±1.3 [a]
3,5,7-Trihydroxychromone	8.3±0.3	42.5±1.6 [b]
Trolox	5.4±0.2	21.5±0.8

IC_{50} value was defined as the concentration of 50% superoxide anion radical inhibition and calculated by linear regression which was analyzed by Origin 6.0 professional software. Means values with different superscripts in the same column are significantly different ($p<0.05$).

ABTS⁺•-scavenging assay

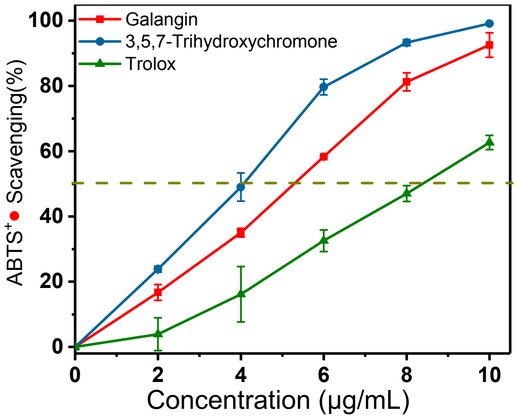

Figure S5: The dose response curves of galangin and 3,5,7-trihydroxychromone in ABTS⁺•-scavenging assay. Each value is expressed as mean ± SD (n = 3).

Table S5: The comparison of IC_{50} values of galangin and 3,5,7-trihydroxychromone and positive control in ABTS⁺•-scavenging assay.

	Mean±SD μg/mL	Mean±SD μM
Galangin	5.3±0.1	19.7±0.1 [a]
3,5,7-Trihydroxychromone	4.1±0.1	20.9±0.3 [a]
Trolox	10.8±0.4	43.3±1.7

IC_{50} value was defined as the concentration of 50% superoxide anion radical inhibition and calculated by linear regression which was analyzed by Origin 6.0 professional software. Means values with different superscripts in the same column are significantly different ($p<0.05$).
