# Supplementary material for: A Null B-Ring Improves the Antioxidant Levels of Flavonol: A Comparative Study between Galangin and 3,5,7-Trihydroxychromone
Source: Molecules. 2018 Nov 26;23(12):3083. doi: 10.3390/molecules23123083 (PMC6321095; doi:10.3390/molecules23123083)
Supplement: Supplementary file 1 [file molecules-23-03083-s001.zip › molecules-393628-SI/Suppls/Suppl. 3 Analysis certificates of 3,5,7-trihydroxychromone.pdf]

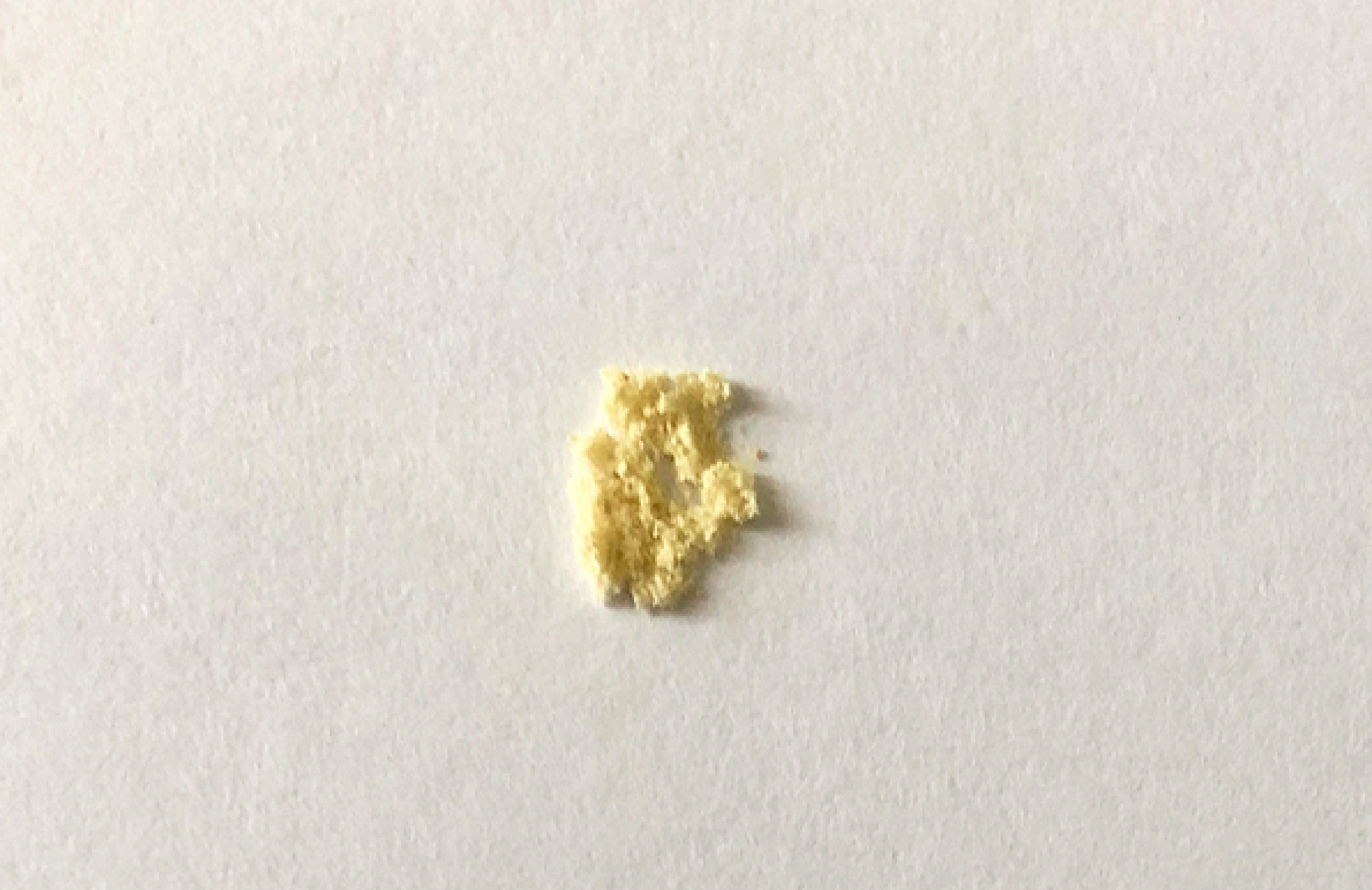

3,5,7-Trihydroxychromone

西力生物
BBP BioBioPha

Natural Product Library for Drug Discovery

CERTIFICATE OF ANALYSIS

BBP No.: BBP04598

CAS No.: 31721-95-6

Chemical Name: 3,5,7-Trihydroxychromone

Molecular Formula: $C_9H_6O_5$

Structure:

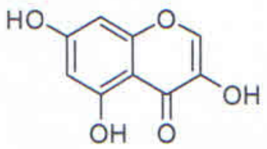

Purity: 98%

Appearance: Yellow powder

Solvent: Methanol, acetone

Exact Weight: 5.1 mg

Storage: Store in a dark place under the temperature of 0−4 °C

Intended Use: For laboratory use only

Reference: G. X. Yang, Chin Pharm J, 2003, 38(5), 338-340

Warm Notice: When publishing, please cite as: ***chemical name*** was purchased from BioBioPha Co., Ltd. (Kunming, China)

Characterization Data Summary

Analytical Test	Results
Identification by ^{1}H-NMR	Consistent with the above structure
Purity tested by HPLC, ^{1}H-NMR	98%

Authorized Signature:

Date:

BioBio[illegible] Co., Ltd.
2018.08.07
PASS

Tel.: +86 871 6521 7109 Fax: +86 871 6521 5563 E-mail: sales@mail.biobiopha.com

西力生物
BBP BioBioPha

Natural Product Library for Drug Discovery

PRODUCT QUALITY REPORT

Product Number: BBP04598

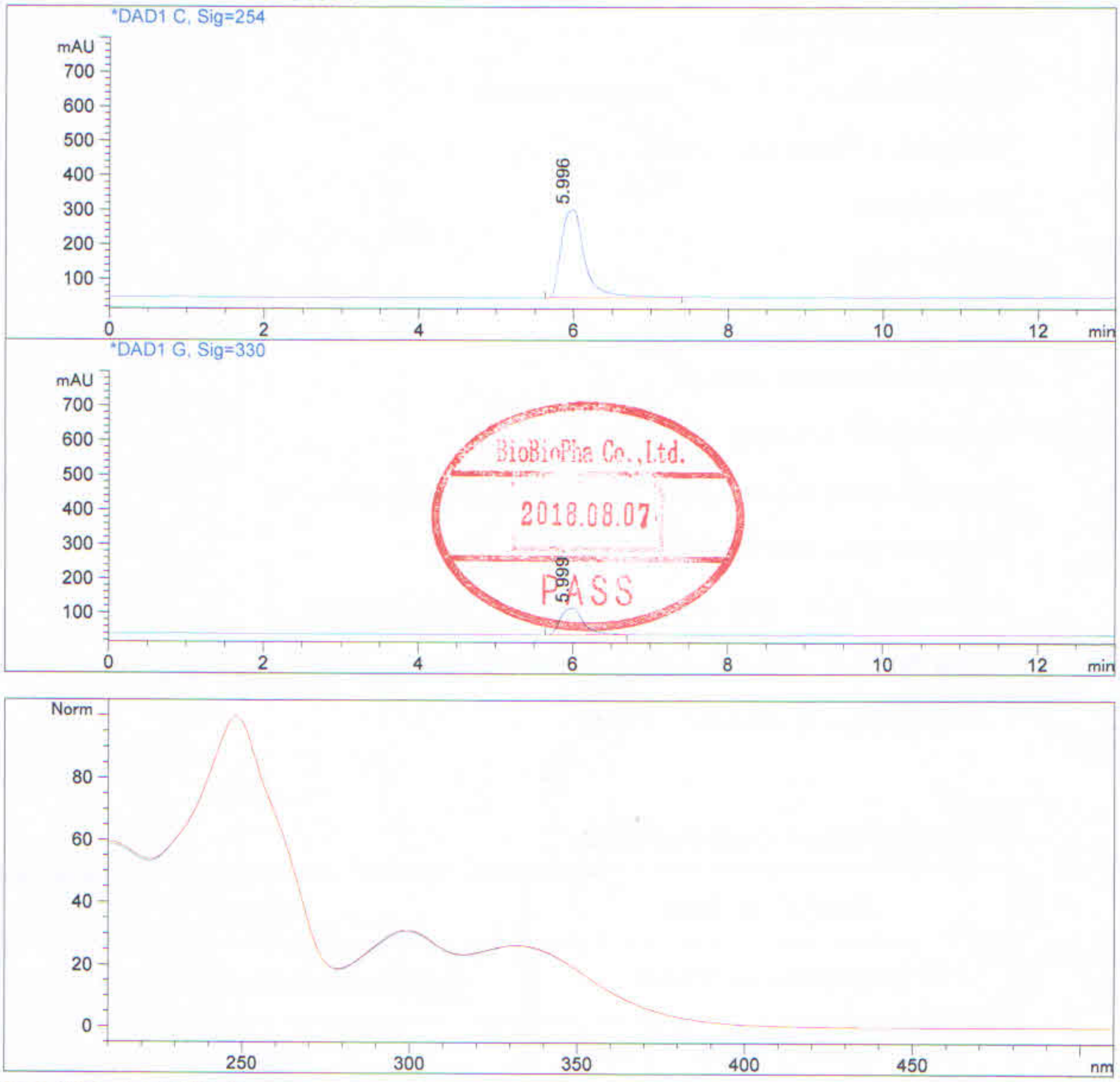

Agilent 1200 series HPLC system
Thermo Hypersil BDS C18 column (5 µm, 4.6 × 150 mm)
20%→100% MeOH in H_2O over 8.0 min followed by 100% MeOH to 13.0 min
1.0 ml/min, 20°C

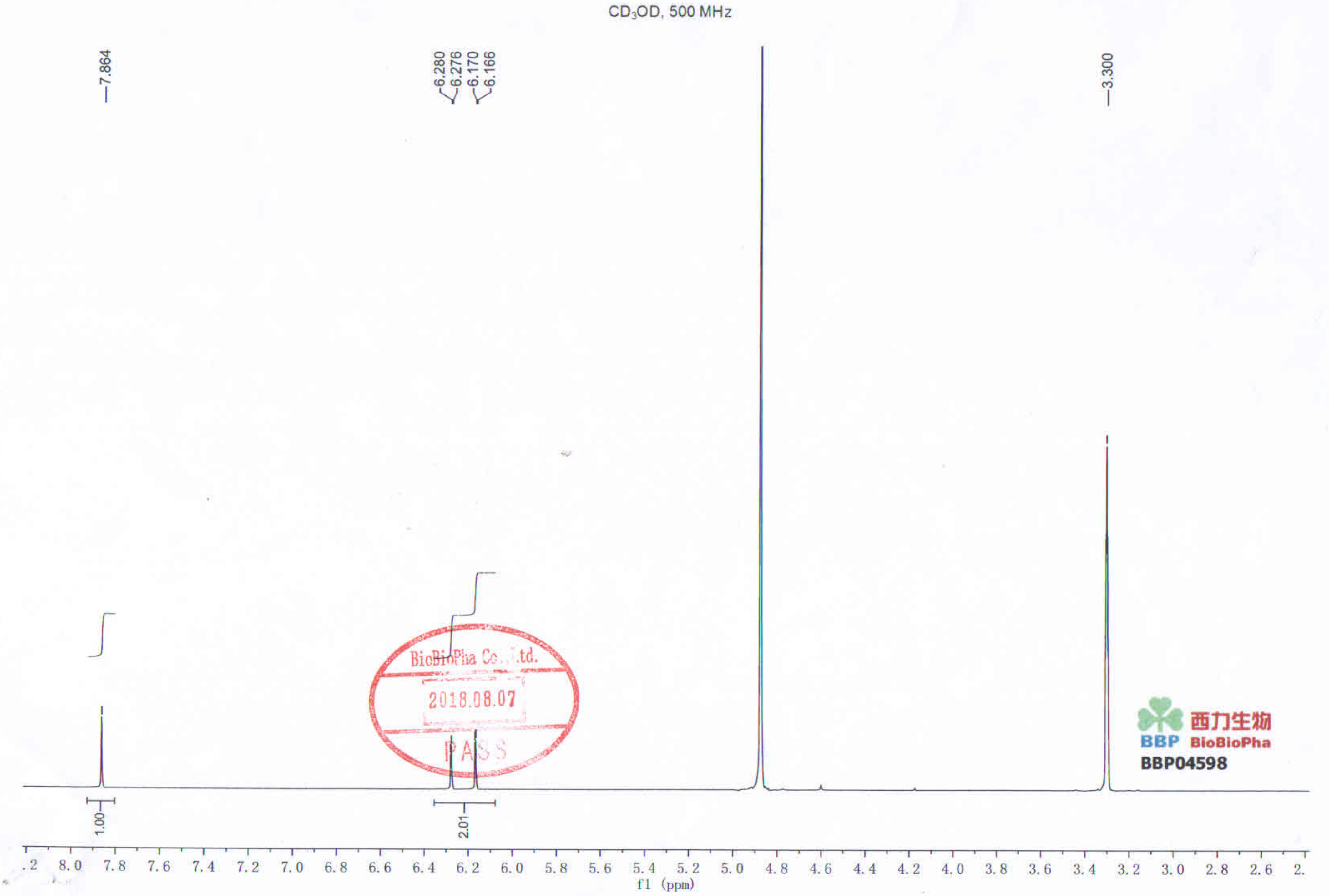

CD$_3$OD, 500 MHz
7.864
6.280
6.276
6.170
6.166
3.300
1.00
2.01
.2 8.0 7.8 7.6 7.4 7.2 7.0 6.8 6.6 6.4 6.2 6.0 5.8 5.6 5.4 5.2 5.0 4.8 4.6 4.4 4.2 4.0 3.8 3.6 3.4 3.2 3.0 2.8 2.6 2.
f1 (ppm)
西力生物
BBP BioBioPha
BBP04598
BioBioPha Co., Ltd.
2018.08.07
PASS
